# Boosting the anti MERS-CoV activity and oral bioavailability of resveratrol via PEG-stabilized emulsomal nano-carrier: Factorial design, in-vitro and in-vivo assessments

**DOI:** 10.1080/10717544.2022.2126028

**Published:** 2022-09-27

**Authors:** Mohamed Y. Zakaria, Islam Zaki, Majid Alhomrani, Abdulhakeem S. Alamri, Osama Abdulaziz, Mohammed A.S. Abourehab

**Affiliations:** a Department of Pharmaceutics and Industrial Pharmacy, Faculty of Pharmacy, Port Said University, Port Said, Egypt; b Pharmaceutical Organic Chemistry Department, Faculty of Pharmacy, Port Said University, Port Said, Egypt; c Department of Clinical Laboratories Sciences, Faculty of Applied Medical Sciences, Taif University, Taif, Saudi Arabia; d Center of Biomedical Sciences Research (CBSR), Deanship of Scientific Research, Taif University, Taif, Saudi Arabia; e Department of Pharmaceutics, Faculty of Pharmacy, Umm Al-Qura University, Makkah, Saudi Arabi; f Department of Pharmaceutics and Industrial Pharmacy, College of Pharmacy, Minia University, Minia, Egypt

**Keywords:** Resveratrol, experimental design, oral bioavailability, anti MERS-CoV activity, inflammatory mediators, oxidative stress

## Abstract

Resveratrol (RSV) is a phytoceutical polyphenolic compound exhibiting a well evidenced wide range of therapeutic activities. Unfortunately, its diminished aqueous solubility and extensive metabolism in gastro intestinal tract (GIT) and liver prohibit its biological activity and systemic availability. Herein the conducted study PEG stabilized emulsomes (PEMLs) were customized to enclose RSV aiming to boost its biological availability and antiviral activity. PEGylating the vesicles not only grant the promoted steric stability of the system but also being beneficial in exaggerating the intestinal permeability and extending the period of circulation of the drug, hence its targeted clinical use. The Investigation of the influence of predetermined variables on the physical characterization of formulae (entrapment efficiency EE%, particle size PS and zeta potential ZP) was implemented utilizing Design Expert® software. (F4) with desirability value (0.772), picked to be the optimal formula, which is fabricated utilizing 35 mg compritol as the lipidic core and 60 mg 1,2-distearoyl-sn-glycero-3-phosphoethanolamine-N-[amino(polyethylene glycol)-2000] (DSPE-Mpeg-2000). The dominance of the F4 relative to RSV dispersion was affirmed by the data acquired from ex-vivo and pharmacokinetic studies. In addition, F4 exhibited significant lower EC_50_ value (0.0127 µg/mL) relative to that of RSV dispersion(0.338 µg/mL) by around 26 times denoting the capability of the formulation to boost the antiviral activity. To a great extent, F4 was able to significantly suppress the inflammatory response and oxidative stress resulted from MERS-CoV infection on comparison with RSV dispersion. Finally, the potentiality of PEMLs as nano-panel with boosted both antiviral and oral bioavailability for RSV could be deduced based on the outcomes mentioned herein.

## 1. Introduction

Nowadays, the evolution of many contagious and fatal viral infections that especially strikes the respiratory tract and predispose to a drastic and lethal consequences, necessitates the elaboration of novel tactics for the preparation of nanotechnology based remedies. Those remedies were capable of efficiently hitting the target without imparting any negative consequences on the host cells. One of the most virulent infection is middle east respiratory syndrome (MERS), or “Camel flu” which initially was recognized in 2012 in Saudi Arabia (Iwata-Yoshikawa et al., 2019; Li and McCray, 2020). Being as corona family member, MERS-CoV can be spread rapidly infecting many people causing grave consequences, various organ collapse and sometimes reaching to death (Iwata-Yoshikawa et al., 2019). The viral resistance owing to those viruses fast genetic mutations can be considered as one of the crucial competitive challenge facing the traditional antiviral remedies.

Stunningly, over the past decades, there was a great attention toward the functionalization of natural supplements as nutriceuticals owing to the capabilities to exert vast pharmacological activities with a broad range of safety profile. Herein, the natural non-flavonoid polyphenol resveratrol (RSV), stilbene derivative, originating from the skin of red grapes and in polygonum cuspidatum. On the basis of well evidenced data, RSV is capable of exerting multiple biological and pharmacological functions as anticancer, anti-inflammatory, antidiabetic, antioxidant and neuroprotectant (Santos et al., 2019; Poonia et al., 2020). Various studies were conducted to explore the antiviral activity of RSV especially against SARS-CoV-2, where its antiviral activity was not restrained against RNA viruses only but extended to conquer a few DNA viruses including poxvirus and polyomavirus DNA ones also (Marinella, 2020; Ramdani and Bachari, 2020; Pasquereau et al., 2021). It is worth mentioning that RSV exhibited its in-vitro anti MERS-CoV activity and induction of viral apoptosis via splitting of MERS-CoV caspase 3 and the suppression of viral nucleocapsid protein translation (Pasquereau et al., 2021).

In addition, it has been mentioned to evoke a crucial anti MERS-CoV activity besides its ability to eradicate its harsh consequences as oxidative stress and inflammatory outcomes (Lin et al., 2017).

Unfortunately, being as a Class II BCS candidate all those activities were prohibited owing to its diminished water solubility besides its liability for extensive metabolism in GIT and liver (Isailović et al., 2013; Santos et al., 2019). Many nano-carrier systems as vesicular system (liposome, niosome), nanoparticles and self-nano emulsifying drug delivery system were implemented in boosting RSV bioavailability but till now no study was conducted adopting emulsomes (9–11). Hence, emulsomes along with other lipidic vesicular system i.e., liposome and niosome could be proposed as a panel to resolve the previously mentioned obstacles that hinder the drug availability and activity (Gregoriadis, 2016). Emulsomes are bilayered lipidic nanoemulsion, they are considered as a compromise between both systems and exhibiting the advantages of both (Aldawsari et al., 2021). Emulsomes are constructed adopting dual crucial components: lipidic core fenced with phospholipid as an outer layer in order to grant vesicular steric stability. In addition, emulsomes can well accommodate the lipophilic drugs and overcome the drawbacks associated with the conventional vesicular system as higher incidence of drug leakage and tendency for agglomeration (Hegazy et al., 2022). Correspondingly, the PEGylation of the surface of emulsomes will aid in achieving extensive circulation duration and boosted steric stabilization (Dubey and Vyas, 2021; Zaki et al., 2021). All the previously mentioned criteria govern the emulsomes as potential carrier for RSV with accentuated oral biovailability.

Herein the reported study, the enhancement in the oral availability and in-vivo antiviral activity was accomplished via fabrication of various formulation of PEGylated emulsomes (PEMLs) considering vast predetermined variables utilizing 2^3^ full-factorial design. Moreover, the picked optimum formula was assessed for its superiority over the RSV dispersion in comparative drug release study. In addition, the domination of the optimum formula was affirmed by ex-vivo and in-vivo studies. Furthermore, the upgraded RSV antiviral activity along with the anti-inflammatory activity owing to the formulation as PEMLs versus RSV dispersion were investigated adopting MERS-CoV challenged animal model.

## 2. Materials and methods

### 2.1. Materials

See supplementary material: S1.

### 2.2. Construction of RSV loaded PEGylated emulsomes (RSV-PEMLs)

With minor modifications, RSV-loaded PEMLs were tailored adopting the thin film hydration approach (Albash et al., 2019). In a rounded bottom flask, 10 mL ethanol: chloroform (2:1) mixture was utilized to dissolve, 20 mg RSV, the lipid core (compritol, or tristearin) in either amounts 35 or 70 mg, along with the stabilizer (coat forming) 30 or 60 mg amounts of 1,2-distearoyl-sn-glycero-3-phosphoethanolamine-N-[amino(polyethylene glycol)-2000] (DSPE-mPEG 2000) and cholesterol (20 mg) where the flask covered with aluminum foil to protect the components from light degradation (Table 1). The organic solvent was completely removed under reduced in rotary evaporator for 30 minutes at 60 °C, where the hydration of the acquired dry film was performed using Phosphate buffer solution (PBS; 10 mL) for 45 min at 60 °C (resembling temperature surpass the transition temperature of the lipid phase) (Tc) (Zeb et al., 2016). The attained PEMLs dispersions were subjected to bath sonicator (Elmasonic, Model LC 60/H Germany) for 10 min at room temperature for subsequent size de-agglomeration. The preparations were preserved at 4 °C in amber-colored glass tubes for later assessments.

**Table 1.** Experimental design outcomes and assessed responses of RSV-loaded PEMLs via 2^3^ full factorial experimental.

Formula	X1 (Lipid core type)	X2 (Lipid core amount in mg)	X3 (DSPE-mPEG 2000 amount in mg)	Y1 (EE%)	Y2 (PS nm)	Y3 (PDI)	Y4 (ZP -mV)
F1	Tristearin	35	30	59.1 ± 2.8	223.4 ± 11.1	0.27 ± 0.04	−30.9 ± 4.4
F2	Compritol	35	30	80.9 ± 3.4	270.9 ± 21.9	0.34 ± 0.06	−36.4 ± 3.6
F3	Tristearin	70	30	70.4 ± 2.1	254.3 ± 24.6	0.26 ± 0.07	−24.5 ± 5.1
F4	Compritol	35	60	75.8 ± 3.7	172.1 ± 19.5	0.31 ± 0.08	−43.6 ± 7.3
F5	Compritol	70	60	84.9 ± 4.2	230.5 ± 21.3	0.47 ± 0.09	−37.8 ± 2.9
F6	Tristearin	70	60	54.6 ± 2.2	201.7 ± 13.4	0.63 ± 0.1	−31.2 ± 5.6
F7	Tristearin	35	60	41.3 ± 2.1	142.5 ± 15.2	0.41 ± 0.05	−36.2 ± 6.3
F8	Compritol	70	30	94.7 ± 3.6	301.8 ± 18.7	0.28 ± 0.04	−35.8 ± 3.7

* NB: 20 mg cholesterol was incorporated in each of the prepared formulae.

### 2.3. Statistical optimization and in-vitro assessment of RSV-loaded PEMLs

#### 2.3.1. Assessment of the entrapment efficiency percentage (EE%)

In triplicates RSV entrapment efficiency percentages (EE%) in the casted PEMLs dispersion was determined: primarily, the dispersion of 1.00 mL aliquot of the RSV-charged PEMLs (enclosing 2.00 mg of the drug) was replenished using distilled water (5.0 mL) followed by agitation for 2 min and finally centrifuged for 1 h (Beckman, Fullerton, Canada) at 15339 g with cooling at 4 °C (Zakaria et al., 2021). The settled vesicles were re-centrifuged for 30 min after being washed off with distilled water for two times. The vesicles were disrupted in methanol using sonication at room temperature for 2 h and the enclosed RSV amounts within the vesicles were investigated by UV-spectrophotometry at λ_max_ 290 nm versus methanol as blank (Negi et al., 2017). EE% values were calculated as follows:

EE% of RSV entrapped = (Amount of RSV enclosed/overall amount of RSV) x 100

#### 2.3.2. Assessments of vesicular diameter, zeta potential and PDI

In glass tube 0.1 mL of each formulated RSV-charged PEMLs was admixed with 10 mL deionized distilled water then vortexed for 5 min. The samples were then introduced to Zetasizer ZS (Malvern Instruments, Malvern, UK) for the assessment of the mean droplet size, zeta potential (ZP) and PDI and they were investigated in triplicate (Mohammed et al., 2020)

### 2.4. Design statistical validity and determination of the optimum RSV-charged PEMLs

Design of experiment and investigation of the outcomes acquired from the alteration in various fabrication attributes were conducted utilizing Design Expert® Version 13 (Stat Ease, Inc., Minneapolis, MN, USA) adopting 2^3^ factorial analyses. The acquired design resulted in the formulation of 8 runs considering the variation in three factors: lipid core type (X1), lipid core amount (X2), DSPE-Mpeg-2000 amount (X3). Meanwhile, EE% (Y1), PS (Y2), ZP (Y3) were chosen as dependent variables. According to the implemented ANOVA statistical analysis, the main impacts of the variables and their significance were outlined. The election of the optimum RSV-charged PEMLs was implemented on the basis of the criteria of the highest ZP, EE% and the minimum droplet size along with the highest desirability value the optimum formula and then it was involved in further assessments (Aldawsari et al., 2021)

### 2.5. The optimum RSV-charged PEMLs formula in-vitro assessment

#### 2.5.1. Optimized PEML formula lyophilization

The optimized RSV-loaded PEML formula solidification was implemented using Lyophilization (Alpha 2–4, CHRIST, Osterodeam Harz, Germany) in presence of cryoprotectant namely Mannitol (5% wt/vol) to prohibit the vesicular damage and aggregation. The optimum formula dispersion was preserved at −80 °C for overnight followed by drying under vacuum for 24 h (Dubey and Vyas, 2021). For DSC characterization, the acquired emulsomal powder was preserved in tightly closed amber-colored glass tubes in a desiccator.

#### 2.5.2. Differential scanning calorimetry (DSC)

The thermal analysis of each of pure RSV along with optimum blank and RSV-loaded PEMLs was assessed utilizing DSC (DSC-50, Shimadzu, Kyoto, Japan). The calibration of the equipment was conducted using Purified indium (99.9%). The thermal behavior of the samples was scanned in a temperature range 20–400 °C by an incremental rate of 10 °C/min (Mohammed et al., 2020).

#### 2.5.3. Transmission electron microscopy (TEM)

TEM (JOEL JEM 1230 Instrument) was utilized for the visualization and determination of the vesicular configuration. The phosphotungstic acid solution (2% wt/vol) stained vesicle’s droplet was then adhered to a carbon grid with copper coating and left to dry to attain a thin film. The acquired copper sheet was visualized by using the TEM (Zaki et al., 2021)

#### 2.5.4. Comparative RSV in-vitro release experiment

1.00 mL of the optimized RSV-charged PEML dispersion (comprising 1.0 mg of RSV) attained after the dilution of 1 mL of the crude formula using 1 mL Sorensen phosphate buffer (pH 7.4) and 1 mL of RSV dispersion (1 mg/mL) were placed in a 2.5-cm diameter 10-cm glass cylinder with a presoaked cellulose membrane fitted at the bottom of the cylinder. Then the glass cylinder was placed in 900 mL dissolution media (Sorensen phosphate buffer, pH 7.4) at 37 °C after being carefully hanged onto the shaft of the dissolution tester (Copley, DIS 8000, Nottingham, UK) revolved at speed of 50 rpm (Abdelbary et al., 2018). The removal of equal volume aliquot samples from the dissolution media at scheduled time intervals was then followed by their replacement using an equal volumes of freshly prepared dissolution medium in order to assure the attainment of a constant volume and constant sink condition. The percentile of RSV released from both optimized RSV-charged PEML relative to that of RSV dispersion were analyzed spectrophotometrically at 290 nm and were assessed in triplicate.

#### 2.5.5. The influence of storage on the physical characteristics of the optimized formula

The optimized formula was preserved for a period of 3 months at each of 4 °C and 25 °C. The samples at 0 and at 3 months, from the optimized formula were withdrawn, then the impact of storage was investigated with respect to the PS, ZP and E.E% values. The significance of the outcomes of the parameters under investigation was examined via one-way ANOVA analysis at a level of *p* < 0.05 (Hegazy et al., 2022).

### 2.6. Ex-vivo RSV permeation experiment

In order to investigate the difference between the drug dispersion and the optimized formula on the RSV permeation capability, the non-everted gut sac technique was adopted (Zakaria et al., 2021). Twelve overnight-fasted male wistar rats (180–150 g) were terminated via cervical dislocation after being subjected to anesthesia using Diethyl ether. The detached small intestines were then sliced into smaller segments of 5-cm length and were rinsed out for removal of any debris utilizing normal saline, finally they were kept under continuous irrigation utilizing Krebs-Ringer solution (∼ 2 mL). The intestinal sacs were formed by closing the bottom of each segment utilizing a thread attached to a blunt ended syringe. A specified volume (that corresponding to 5-mg RSV) of each of the RSV dispersion (prepared using 20 mg of RSV in PBS) and RSV optimum formula was placed in each sac and this end was then closed, subsequently each sac was immersed in 50 mL of Krebs-Ringer solution in 100 rpm stirred organ bath kept at 37 ± 0.5 °C with continuous aeration with an 95:5% oxygen: CO_2_ admix. The amount permeated of RSV was computed in each withdrawn sample at various time interval (15, 30, 45, 60, 75, 90 min) followed by replacement of the withdrawn volume using an equal volumes of fresh Krebs-Ringer solution. The amounts of RSV in the receptor solution were computed utilizing Hitachi LaChrome Elite HPLC fitted with L-2130 pump with built in degasser, L-2455 photo diode array detector (DAD), L-2300 column oven, a Model Series L-2000 organizer box and Rheodyne 7725i injector with a 20 mL loop, Column: reversed phase C18, 4.6 × 100 mm, 5 μm, Xterra, USA, utilizing mixture of methanol and potassium dihydrogen phosphate buffer (pH 3.8) in a ratio 40%:60% vol/vol as a mobile phase which flow at a constant rate of 1.0 mL min^−1^ and the drug was detected at the wavelength 320 nm (Negi et al., 2017).

The apparent permeability (Papp) of the optimized RSV-charged PEML versus RSV dispersion was computed adopting the following formula:

$$\text{Papp}\;=\;\left({\text{cm}\;\text{min}}^{-1}\right)\;F/A*C_{0}$$

where F is permeation flux that indicate the amount of RSV permeated across the membrane per unit time, C_0_ is the initial concentration, and A is the total surface area of the ileum.

### 2.7. Cellular anti-viral activity investigations

#### 2.7.1. MTT cytotoxicity assay (TC_50_)

Tiny modification was adopted during the assessment of the cytotoxic activity of the samples under investigation on Vero E6 cells utilizing dimethylthiazol-2-yl)-2,5-diphenyltetrazolium bromide (MTT) (Zhang et al., 2020). The tested samples were diluted via Dulbecco’s Modified Eagle’s Medium (DMEM). In 96-well plates (100-µL/well at a density of 3 × 10^5^ cells/mL) the cells were assembled cells and then incubated in 5%CO_2_ for 24 h at 37 °C. Subsequently, the investigated compounds in different concentrations were incubated with the cells for an extra 24 h and each experiment was repeated three times. The supernatants were then detached from the cell monolayers and rinsed 3 times with sterile phosphate buffer saline (PBS). The harvested cells were allowed to be stained with MTT solution (20 µL of 5.0 mg/mL stock solution) and incubated for 4 h at 37 °C in each well followed by aspiration of the medium. The incorporation of 200 µL acidified isopropanol (0.040 M HCl in absolute isopropanol = 0.073 mL HCL in 50 mL isopropanol) in each plate in order to dissolve the attained formazan crystals. Multi-well plate reader was involved for the determination of the formazan solutions absorbance’s at λ_max_ 540 vs the 620 nm reference wavelength. The cytotoxicity percentile relative to the untreated cells was estimated utilizing the following equation:

% cytotoxicity= (absorbance of cells without treatment−absorbance of cells with treatment) x100/(absorbance of cells without treatment)

From the curve of cytotoxicity percentage versus concentration, (TC50) was deduced (Joshi et al., 2016).

#### 2.7.2. Plaque inhibition assessment

MERS-CoV viral titers were determined adopting plaque loaded on Vero E6 cells assay. As previously conducted, in six-well plates Vero E6 cells (10^5^ cells/mL) were cultivated for 24 h at 37 °C (Zakaria et al., 2021). 10^3^ PFU/well attained via the dilution of MERS-related coronavirus isolate NRCE-HKU270 (Accession Number: KJ477103.2) were then incubated for 1 h at 37 °C with the safe concentration of the samples under investigation. The samples of the investigated formulae admixed with 2% agarose and 3 mL DMEM were placed with cells challenged with virus (100 µL/well) for1 h as a proper contact time for viral adsorption. The plates were allowed to be solidified and incubated at 37^0^ C for 3 to 4d till the evolution of viral plaques. The cells were stained with 0.1% crystal violet in distilled water after being subjected to 10% Formalin for 2 h. On another hand, the wells that were determined as control (untreated virus) were incubated with Vero E6 cells then, the relative percentage of decline in plaques count to that in the control wells was estimated as follows:

"% inhibition= (viral count (untreated) − viral count (treated)) x 100/viral count (untreated) "

### 2.8. Pharmacodynamics assessment of the selected RSV-charged PEML

#### 2.8.1. Experimental animals and MERS-CoV viral infection

The Biological Production Unit (BPU) of the Theodore Bilharz Research Institute (Giza, Egypt) was the source of the forty 10-week-old female C57BL/6 mice of weight 150 ± 10 g. The accommodation of the experimental mice for seven days was in standard polypropylene cages and kept under optimum laboratory circumstances concerning light with 12-h of dark/light cycles, temperature and humidity. The mice were allowed to freely access the water and diet *ad libitum* aiming to reduce the variation (Mohammed et al., 2020). The experimental protocol was approved by Faculty of pharmacy, Port Said University. The utilization and handling of animals in all studies obeyed the EU directive 2010/63/EU. Half tissue culture infectious dose (TCID50) of MERS-CoV (10^5^ pfu) enclosed in 50 µL of viral solution enclosing was intranasally inoculated in the animals under investigation (Hong et al., 2020). The experimental animal groups (10 mice for each) were kept under regular monitoring of their body weight and the rate of their survival. The random assignment of the animals into different groups was adopted and consecutively they attained different dosage regimen (all doses in PBS) as follows:

Uninfected (Control) Group 1: received (2 mL/kg) saline intraperitoneally (i.p.) once followed by daily oral administration of saline at dose of 5 mL/kg.
MERS infected (untreated) Group 2: the infection achieved via intranasal inoculation of 10^5^ pfu/50 μl of virus
RSV suspension treated Group 3: post infection, an oral daily dose of 10 mg/kg utilizing infant feeding syringe (Lin et al., 2017) of RSV suspension was administered for 6 d.
RSV-PEML treated Group 4: post infection, an oral daily dose of 10 mg/kg (Lin et al., 2017) of RSV PEML was administered utilizing infant feeding syringe for 6 d.

#### 2.8.2. Viral titer investigation in acutely infected lungs adopting plaque assay

The animals were subjected to urethane (1.6 g/kg) at day 3 and day 6 for anesthesia, succeeding their viral infection, they were terminated by intraperitoneal overdose of sodium pentobarbital (30-50 mg/kg) (on day 6). The excised lungs were immediately gathered and rinsed with normal saline followed by drying using tissue paper. Furthermore, the weights of the harvested lungs were recorded, then they were supplemented via 0.1% SDS, Roche complete ULTRA Tablet, physiologic saline (1.0 mL) and protease inhibitor (20 mM Tris-HCl, 150 mM NaCl, 1% Triton X-100). Afterwards, the harvested lungs were homogenized utilizing a glass tissue homogenizer (MPW-120 homogenizer, Bitlab, Shanghai, China), and finally centrifuged for 10 min at 7826 g. The harvested aspirates were preserved at −20 °C or at −80 °C for subsequent analysis (Coates et al., 2018). The lung homogenates were serially diluted using DMEM for subsequent infection with Vero E6 cells at 37 °C for 2 h. In 12-well plates, the attained Vero monolayers were cultivated and then challenged with serially diluted infectious virus contained in the lung homogenate (200 μl/well) and the assay was conducted as previously described.

#### 2.8.3. Estimation of BALF levels of IL-6, TNF-α, superoxide dismutase (SOD) and plasma level of glutathione (GSH)

See supplementary material: S2.

### 2.9. Pharmacokinetic study

In accordance with the research protocol approved by Cairo University, Institutional Animal Care and Use Committee (ICU/I/F/85/25): Twelve male Wistar albino rats (220-250 g) were accommodated standard polypropylene cages and were preserved under optimum laboratory circumstances of free accessibility to the standard laboratory water and diet ad libitum, temperature, humidity, and light. The rats were subcategorized into two groups each of six. Group (I) animals received an oral dose of RSV dispersion enclosing 20 mg/kg of RSV dispersion in PBS whose PS was 697.4 nm ± 42.1 and PDI 0.813 ± 0.12, while, Group (II) animals received an oral dose of what corresponding to 20 mg/kg of RSV contained in the dispersion of optimum RSV-charged PEML formulation (Poonia et al., 2020). A day before the administration of the investigated formulae both groups were kept overnight fasted. From the tail veins of each animal, blood samples each of (0.50-mL) were captured at different time intervals (0.5, 1, 2, 4, 6, 8, 12, 18 and 24 h) and were preserved into EDTA-coated tubes. The segregation of the plasma was conducted via centrifugation for 10 min at 959 g. afterwards, the prepared samples were preserved at −80 °C up till they were subjected for further analysis. The concentrations of residual RSV in the plasma were assessed utilizing the HPLC methodology described later.

The considered RSV pharmacokinetic parameters for each rat in the two groups: the one administered the RSV-charged PEML and the group administered RSV dispersion were assessed adopting non-compartmental pharmacokinetic models (Poonia et al., 2020) with the Thermoscientific Kinetica® software version 5 (Cherry Street, Philadelphia, USA). The discrimination between C_max_ and AUC_0–24_ of both types of preparations was governed utilizing One-way ANOVA statistical tests. Moreover, the appraisal of the gap between the median s of Tmax was employed via the non-parametric signed-rank test (Mann–Whitney’s test) with the statistical software of SPSS version 14 and *p* < 0.05 was the level of significance (Albash et al., 2019).

## 3. Results and discussion

### 3.1. Experimental design statistical evaluation

The elaboration of 2^3^ full factorial design owing to assess the confounding variables impact on the prospected responses. Table 1 displayed the emerged 8 experimental runs and their relative characterizations EE%, PS and ZP. The design space appropriateness could be governed by the proper model precision value (Ahmed et al., 2020; Zakaria et al., 2021). All the dependent responses exhibited reasonable precision value of ratio exceeding four (Table 2). Moreover, all dependent variables affirmed an acceptable harmony between their predicted and adjusted R^2^ as the difference between their values did not exceed 0.2 (Table 2). Table 2 also revealed that the adjusted R^2^ values were consistent with the predicted R^2^ values in all the dependent variables (Mosallam et al., 2021).

**Table 2.** The outcomes of the statistical and factorial investigation of 2^3^ design and related formulae of RSV-loaded PEMLs with the predicted, observed responses and deviation percent of the optimum formula (F4).

Responses	EE (%)	PS (nm)	ZP (mV)
R^2^	0.976	0.971	0.949
Adjusted R^2^	0.959	0.948	0.912
Predicted R^2^	0.906	0.883	0.8
Adequate precision	20.5	19.1	14.6
Significant factors	X1, X2, X3	X1, X2, X3	X1, X2, X3
Observed value of the optimal formula (F4)	75.8	172.1	−43.6
Predicted value of the optimal formula (F4)	72.1	183.5	−43.3
Absolute deviation %	4.9	6.6	0.7

### 3.2. The fabrication variables impact on EE%

**Figure 1.**
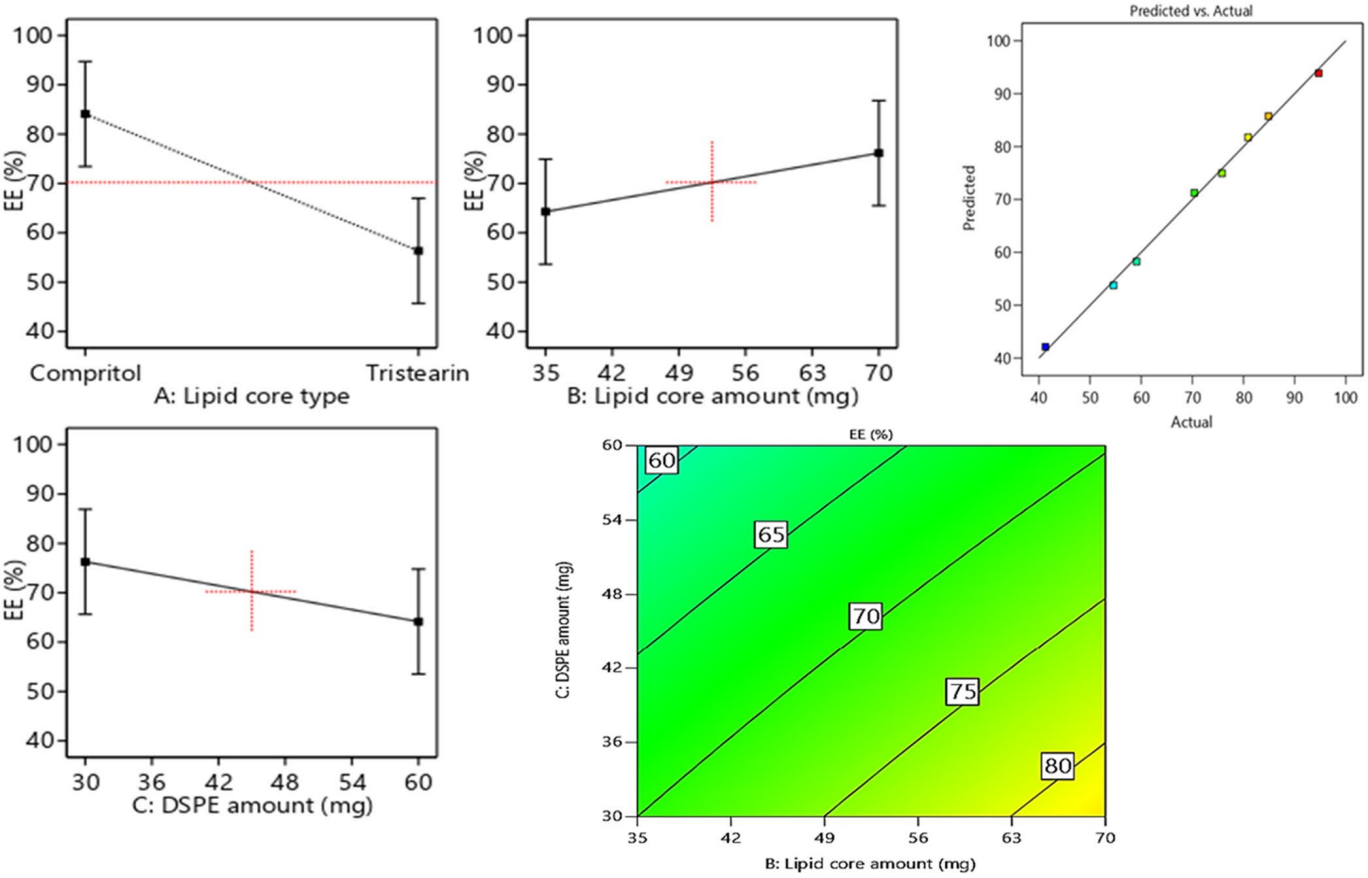
Linear and contour plots revealing the impact of fabrication variables on EE% and predicted versus actual correlation plot.

The assessment of EE% is very propitious in highlighting the lodging capability of the vesicles to RSV. The enclosed percentage of RSV within the vesicles ranged from 41.3% ± 2.1% to 94.7% ± 3.6%. Figure 1 revealed the linear and contour plots of all variables impact: lipid core type (Factor X1), Lipid core amount (Factor X2), DSPE-Mpeg-2000 amount (Factor X3) on the EE% and it can be summarized as follows.

Concerning the lipid core type (Factor X1), the outcomes of ANOVA analysis revealed that the EE% of compritol based formulae significantly (*p* = 0.0004) surpassed the EE% values of those composed of tristearin; this may be attributed to the disparity in the hydrocarbon chain length between compritol a blend of different glyceryl esters, including the diester glyceryl dibehenate, of the C-22 saturated fatty acid, behenic acid and the saturated tri acyl ester with stearic acid C-18 (Aburahma and Badr-Eldin, 2014). Fundamentally, the increase in the solid lipid hydrocarbon chain accompanied by a prominent decline in hydrophilic-lipophilic balance (HLB) values which in turn will promote the lipids solubilization capability toward the lipophilic molecules as RSV. Moreover, the probability of creation of defected and less organized crystalline configuration was higher in case of compritol as it is composed of a blends of different types of acyl glycerols (mono-, di- and triacylglycerols) versus the symmetric monoacid triacylglycerols in case of tristearin which primed to extra voids for RSV accommodation, hence a higher entrapment of RSV can be denoted.

ANOVA results also revealed that the increment in the lipid core amount (Factor X2) from 35 mg to 70 mg results in significant(*p* = 0.0092) improvement in the endorsement of RSV, this came in accordance to that previously mentioned in (El-Zaafarany et al., 2016). The increment in the lipid amount assures the availability of enough lipid for RSV accommodation. Also the increment in the lipid amount predispose to elevation in the viscosity of the vesicular system which in turn can prohibit RSV diffusion throughout the lipophilic core leading to increased drug enclosure (Albash et al., 2019).

With respect to the influence of increasing the amount of DSPE-mPEG 2000 (FactorX3) from 30 mg to 60 mg on EE%, a significant (*p* = 0.0085) in EE% values could be noticed, this may be attributed to the increased possibility of interference with the vesicular structure and development of pores within the membranes (Albash et al., 2019). Additionally, the solubilization of RSV will be increased instead of being encapsulated in the vesicles on increasing the amount of DSPE-mPEG 2000. Hereby, the previously mentioned reasons were thought to be behind the noticed decline in RSV EE% on increasing the amount of DSPE.

### 3.3. PDI and the influence of the compounding variables on vesicular size (PS)

#### 3.3.1. PDI

The extent of homogeneity and the scattering of the particles around the mean PS can be governed by PDI value, hence, monodispersity can be deduced for values approaching zero, on another hand, for values approaching 1 polydispersity (PS) can be denoted. The RSV-loaded PEMLs PDI values revealed in Table 1 was in range of 0.26 ± 0.07 to 0.63 ± 0.1. Thus, the trend of PDI values of RSV-loaded PEMLs align conveniently toward polydispersity (Stetefeld et al., 2016). Unfortunately, the investigated factors exploited a non-significant influence on PDI with p values of 0.589, 0.345 and 0.082, respectively.

#### 3.3.2. PS

**Figure 2.**
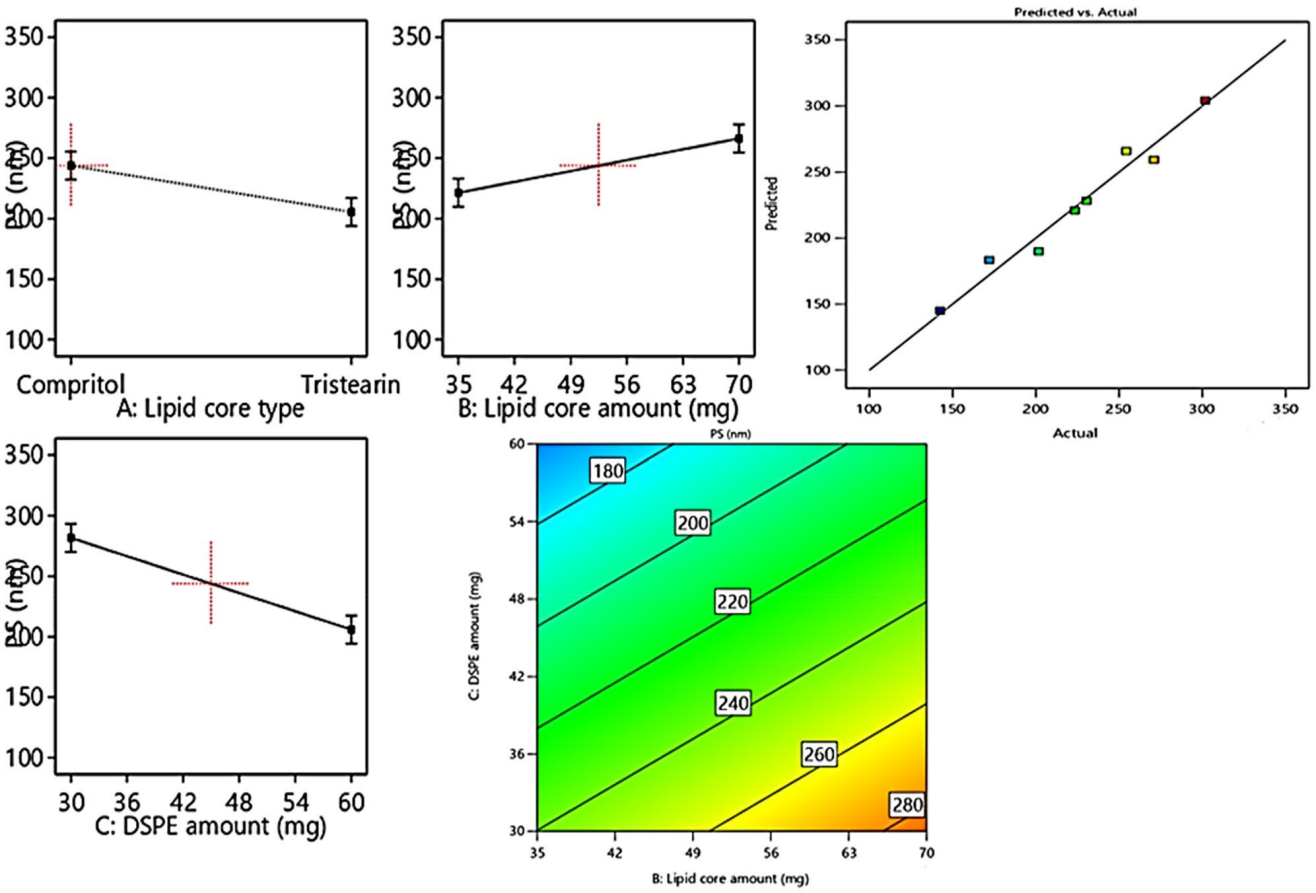
Linear and contour plots revealing the impact of fabrication variables on PS and predicted versus actual correlation plot.

In general, the particle size of the drug exhibits a great impact on the overall fate of the drug in the systemic circulation besides its the impact on the drug’s activity, thus the therapeutic activity will be promoted on suppressing the size owing to the boosted intestinal permeation and extended drug residence duration. From Table 1, it can be depicted that the fabricated formulae average particle size of the ranged from 142.5 ± 15.2 to 301.8 ± 18.7 nm. From the results of ANOVA, it can be deduced that the three variables of the, Factors X1–X3 possessed a significant impact on PS, and represented graphically as the linear and contour plots (Figure 2). The influence of variables on the PS can be individually discussed as herein below.

For the lipid core type (Factor X1), the formulae composed of compritol exhibited significant (*p* = 0.01) larger PS relative to those composed of tristearin. This may be attributed to the greater fatty acid chain length involved in compritol relative to those of the stearic acid involved in tristearin; besides, the lower EE% of tristearin formulae leading to diminishing their PS.

Regarding (Factor X2): the lipid core amount, the increment in the lipid core amount results in a significant elevation (*p* = 0.0058) in PS, which may be owing to the increased tendency of the particle to agglomerate due to the subsequent elevation in the viscosity of attained formulated emulsion on utilizing greater amount of lipids (Zakaria et al., 2022).

Concerning (Factor X3), the higher amount of DSPE–mPEG-2000 resulted in a significant (*p* = 0.0008) suppression in PS, owing to the increased steric bulkiness, hence steric hindrance achieved on increasing the content of PEG attained on increasing the amount of DSPE-mPEG 2000 predisposed to suppress the particle agglomeration tendency and restrain the vesicular aggregation probability, thus constrains the particle size growth and boosts the stability of the system (Zaki et al., 2021).

### 3.4. The influence of the compounding variables on ZP

**Figure 3.**
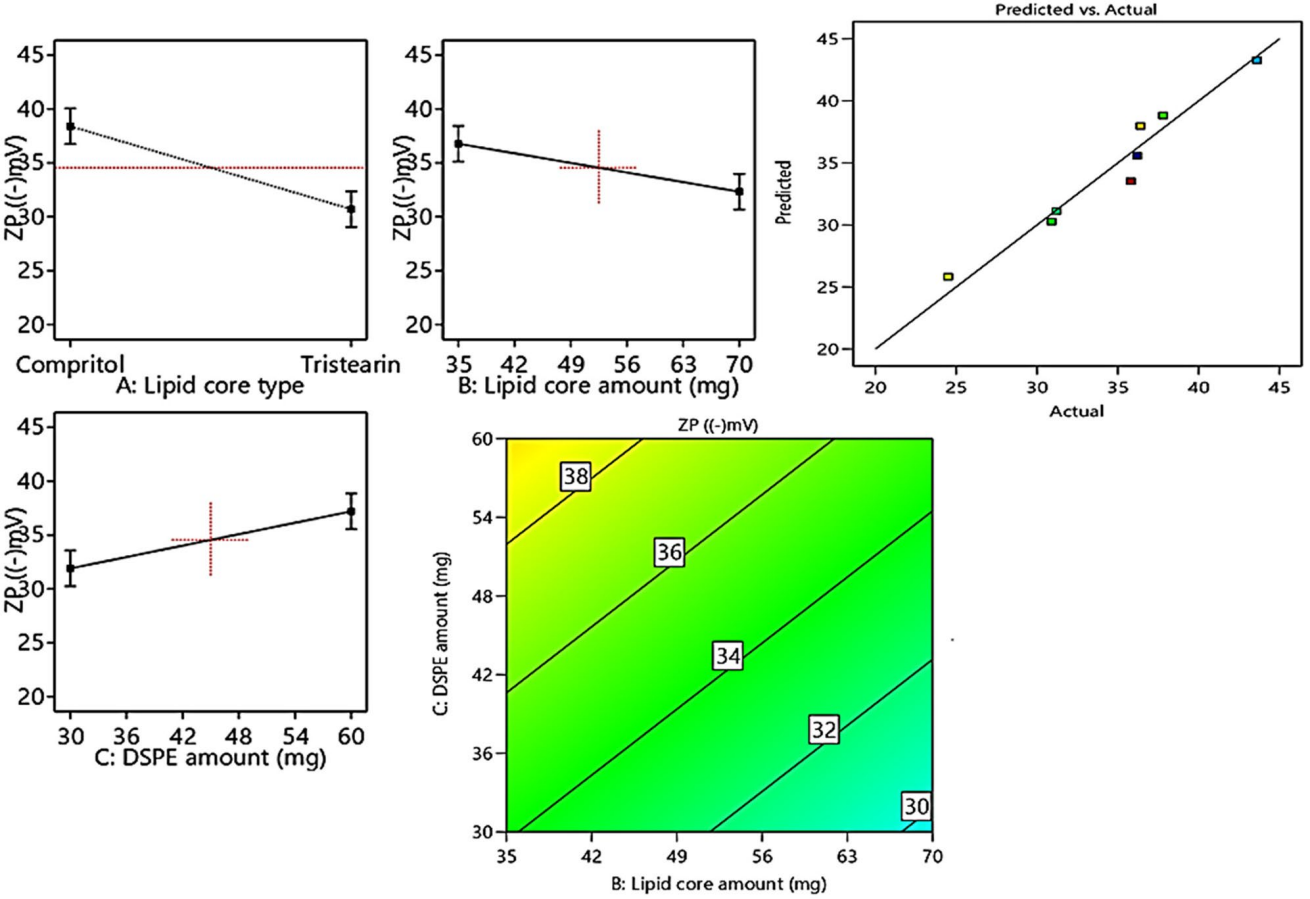
Linear and contour plots revealing the impact of fabrication variables on ZP and predicted versus actual correlation plot.

A clue on the stability of the vesicular system can be attained by the assessment of Zeta potentials (ZPs). Values of ±30 mV are adequate to impart proper electrostatic repulsion forces between the vesicles, which in turn will boost the vesicular electrical stability (Hegazy et al., 2022). Table 1 revealed the investigated ZP values of the prepared PEMLs, which were in range of −24.5 ± 5.1 to −43.6 ± 7.3 mV. The ANOVA outcomes also denoted that all the investigated values conveyed significant influence on ZP as displayed in the linear and contour plots (Figure 3). Their influence can be encountered as follows.

Regarding (Factor X1): type of lipid core alteration predisposed to a significant positive impact (*p* = 0.003) on ZP. As the formulae comprising compritol exhibited higher ZP values than those of tristearin, this may be attributed to the greater engulfment for RSV in compritol –based formulae resulted in the allocation of higher numbers of hydroxyl groups associated with RSV (i.e., polyphenolic compound) in the vesicles. Moreover, the higher amount of lipid (Factor X2) will significantly (*p* = 0.0122) elevate the ZP values owing to the dispersion of more RSV (negatively charged compound) through the lipid core which will increase the net electronegativity of the vesicles.

To a great extent, the inclusion of higher amount of DSPE–mPEG-2000 (Factor X3) predisposes to significant (*p* = 0.0113) higher ZP values. This may be attributed to the alignment of the anionic DSPE–Mpeg (attained from the anionic essential groups involved in its structure) in the form of electronegative coat that wrap the lipid core will densify the electronegativity at vesicular surfaces (Muthu et al., 2011). According to Muthu et al., who investigated the higher electronegativity of the vesicles wrapped with PEG coating relative the uncoated carriers (Muthu et al., 2011; Zaki et al., 2021).

### 3.5. Statistical validation, optimization study and validation of the optimal RSV-loaded PEMLs

**Figure 4.**
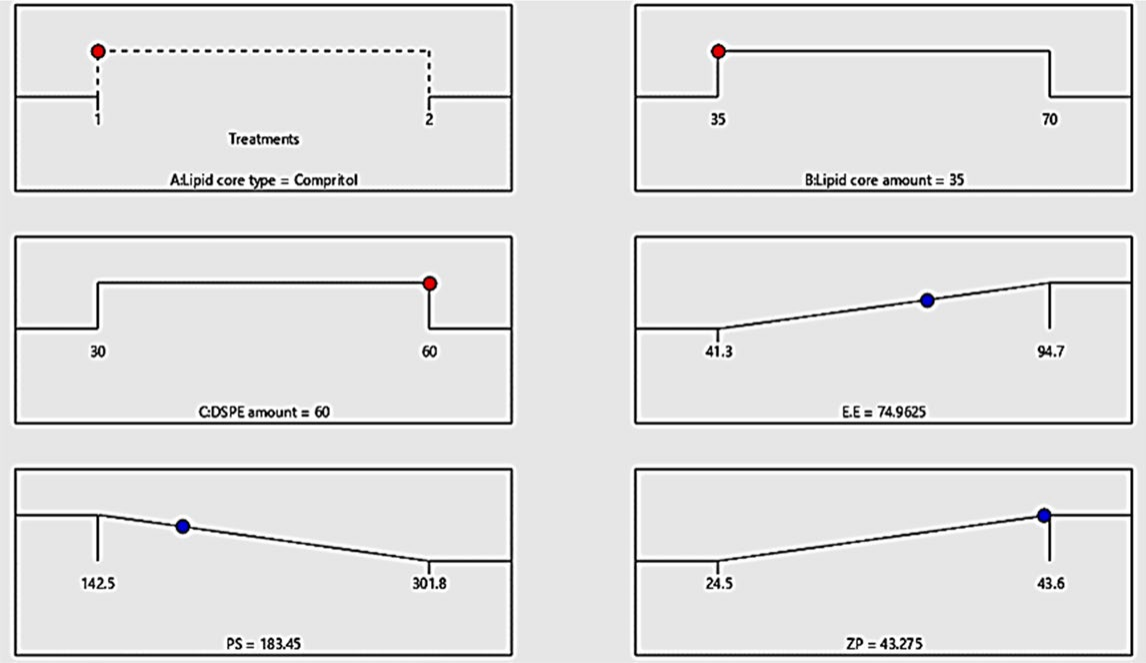
The optimum criteria of the formulation variables for RSV-loaded PEMLs formulae with the projected value of each measured formulation parameter are exploited by optimization ramps for the investigated independent variables.

As subsequent to the aforementioned outcomes, the optimum formula could be selected post the investigation of the dependent variables outcomes adopting Design expert software. F4 constructed from compritol as lipid core utilized in amount of 30 mg with 60 mg of DSPE–mPEG-2000 was proclaimed as optimal formula exhibiting the highest desirability value among the tested formulae (0.772). Additionally, the discrepancy percentage of the predicted from the observed values was calculated for each variable: EE%, PS and ZP and collectively added to Table 2, where it can be denoted that the all the values of the percentage of discrepancy for all the investigated variables were lower than 10% affirming the validity and the appropriateness of our design in the investigation and the analysis of the data (Anwer et al., 2022). Figure 4 disclose the optimum criteria of the optimized RSV-loaded PEMLs (F4).

### 3.6. In-vitro investigations of the optimum RSV-loaded PEML formula

#### 3.6.1. Differential scanning calorimetry (DSC)

**Figure 5.**
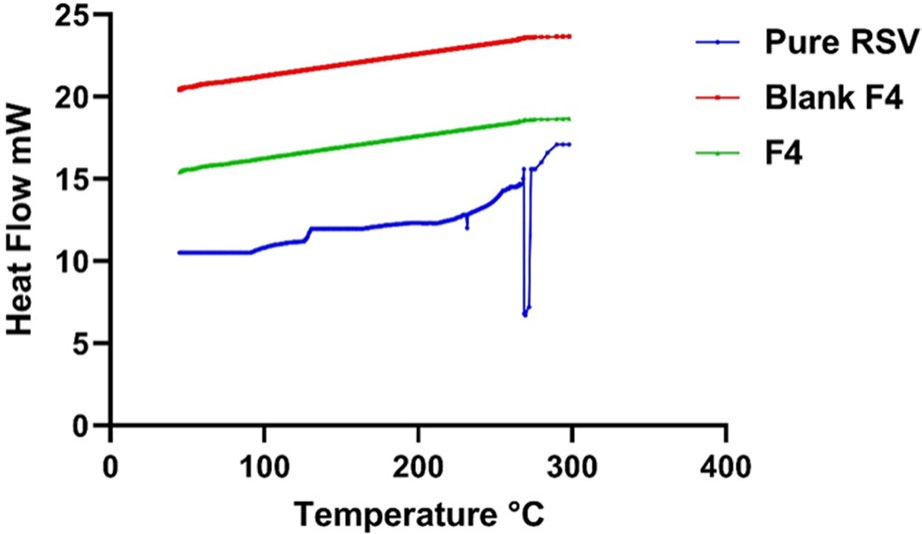
DSC traces: blue—pure RSV; red and green—blank F4 and RSV-loaded optimum formula F4.

In an attempt to assess the alteration physical state and the extent of crystallinity of RSV, DSC study was employed on the pure drug and post being loaded on the nano-carriers. DSC attitude of the pure RSV, blank lyophilized formula relative to that of the lyophilized RSV-loaded optimum PEMLs (F4) are represented in Figure 5. RSV impart a well distinct pointed endothermic peak at 270.2 °C which is corresponding to RSV melting point (Isailović et al., 2013). On another hand, the DSC curves of the lyophilized blank formula and F4 revealed the vanish of any distinctive peak of either RSV or for the components involved in the vesicular fabrication. This assured the complete conversion of the drug and the formula other components to amorphous phase instead the crystalline one and hinting to the efficiency of the drug engulfment into the vesicles.

#### 3.6.2. Transmission electron microscope (TEM)

**Figure 6.**
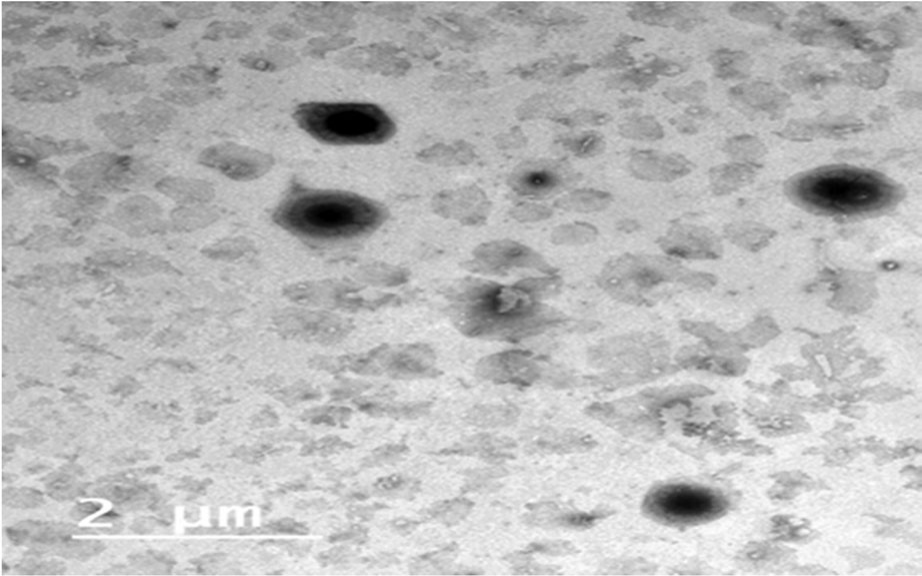
The image of TEM of the picked optimized PEML (F4).

Figure 6 revealed the TEM of F4 which conveyed the rounded shaped vesicular drug carrier lacking any abnormality. In addition, fader borders of the vesicles figure to the alignment of the PEG at the borders of the vesicles (Zakaria et al., 2022). The image did not expose any the arise of any drug crystals which came in accordance of DSC results the assure the total engulfment of RSV inside the vesicles (Abdelbary et al., 2018)

#### 3.6.3. Comparative RSV in-vitro release experiment

**Figure 7.**
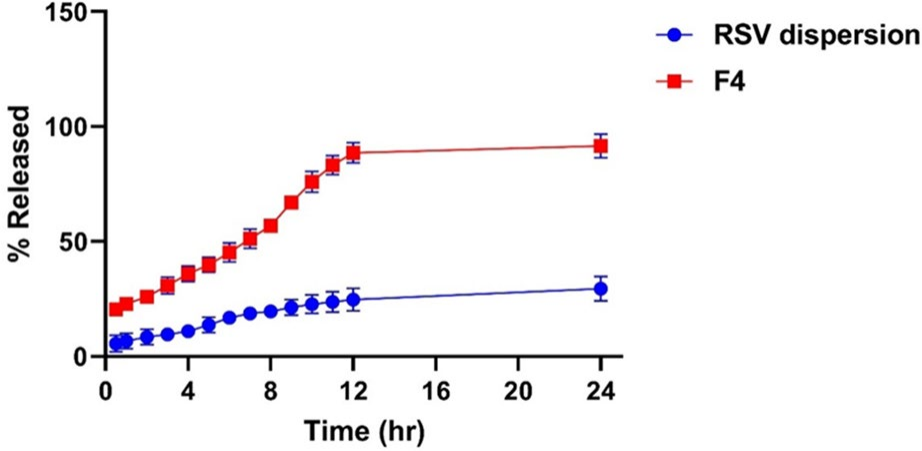
% of RSV released ± SD from the optimized PEML (F4) relative to that of RSV dispersion.

The investigation of the in-vitro release profile of RSV from the optimized PEMLs (F4) was employed to figure out the extent of drug release, solubilization and stability configuration after being incorporated into the vesicles. Figure 7 demonstrated the retarded and progressive release of RSV from the optimized formula (F4) over 24 h, where the amount of RSV released cumulatively from F4 was 91.4% ± 4.2% relative to 27.8% ± 3.2% for the RSV dispersion (*p* < 0.05). This can be justified by the impact of the drug encapsulation in the vesicles which serve as a drug pool for restrained drug release pattern superseded an initial burst phase. While, in case of RSV dispersion the high possibility of drug agglomeration and precipitation owing to its poor wettability and solubility may be behind its restrained release. Moreover, the PEGylated sheath surrounding the vesicles which predispose to higher drug solubilization capability and elevated RSV releases as a consequence of inclusion of DSPE in formulation which will impart increased hydrophilicity and solubilization power for the drug due to its PEG moieties (Alemi et al., 2018).

#### 3.6.4. The influence of storage on in-vitro physical criteria of the optimal formula

From supplementary material Table S6, it can be deduced that the short term preservation of F4 for 3 months at 4 °C and at 25 °C did not impart significant (*p* > 0.05) alterations to the parameters under assessments: EE%, PS and ZP. The steric hindrance acquired as a consequence of the involvement of PEGylated lipid aided in prohibition of drug leakage and vesicular agglomeration, thus promoting the vesicular stability (Zakaria et al., 2021).

### 3.7. Ex-vivo RSV gut permeation study

**Figure 8.**
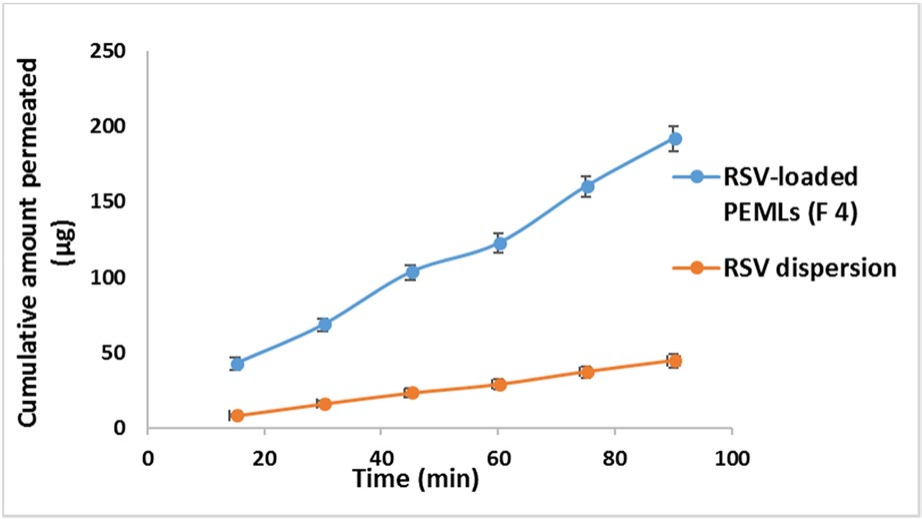
Comparative ex-vivo study results revealing the cumulative amount of RSV permeated (µg) ± SD from RSV-loaded F4 PEML versus the pure RSV dispersion.

RSV intestinal lavage was assessed adopting the non-everted gut sac method for RSV dispersion relative to RSV-loaded optimum formula (F4) in an attempt to prospect the in-vivo attitude, kinetic and availability of the drug to biological system. The superiority of F4 flux J_max_ of F4 2.1 ± 0.084 μg/cm^2^/min after 90 minutes of the experiment can be noticed compared to the flux of RSV dispersion (J_max_ of RSV dispersion = 0.55 ± 0.022 μg/cm^2^/min). The apparent permeability coefficient (APC) of RSV was 2.28 × 10^−4 ^cm/min, on another hand that of the F4 formula was elevated significantly (*p* < 0.05) by around 3.8 times to 8.64 × 10^−4 ^cm/min. Figure 8 revealed the dominance of F4 relative to the RSV dispersion throughout the experimental time owing to its diminished PS and total inclusion in the vesicles which facilitate and fasten its diffusion crossing the intestinal membranes. This may be due to the ability of the drug to by-pass the pitfalls that prohibit its absorption upon encapsulation in the lipidic carrier (Sallam and Marin, 2015).

### 3.8. Investigation of in-vitro anti-MERS-CoV activity

#### 3.8.1. MTT cytotoxicity assay (TC50)

**Figure 9.**
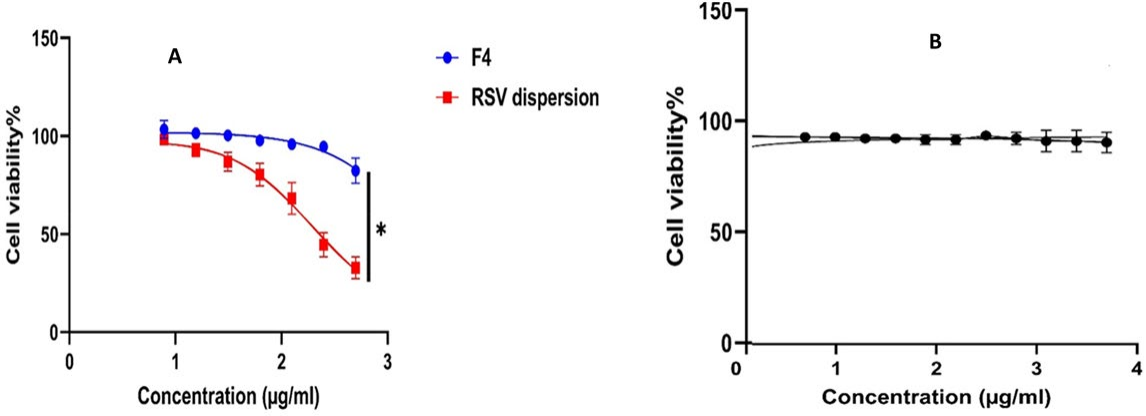
Cellular viability results of (A) RSV dispersion (red); RSV-loaded PEML (F4) (blue) and (B) plain PEML (F4) (Black). *Denotes significance level at *p* < 0.05.

A promising antiviral drug should compromise between the antiviral activity at lower concentration, meanwhile, imparting cytotoxicity on using higher concentrations. The host cell cytotoxicity of F4 was found to be (9.2 µg/mL) and was (2.5 µg/mL) for the RSV suspension (Figure 9); thus F4 exhibited dominant (*p* < 0.05) safety profile by around 3.7 folds relative to RSV dispersion. However, the Plain F4 exhibited cell viability exceeding 90% which assure the safety of the formulation ingredients. Moreover, the assessment of TC50 will outline the safe range of concentrations for being employed in further investigations besides, granting the exclusion of any host cell toxicity throughout the treatment course.

#### 3.8.2. Plaque assay

**Figure 10.**
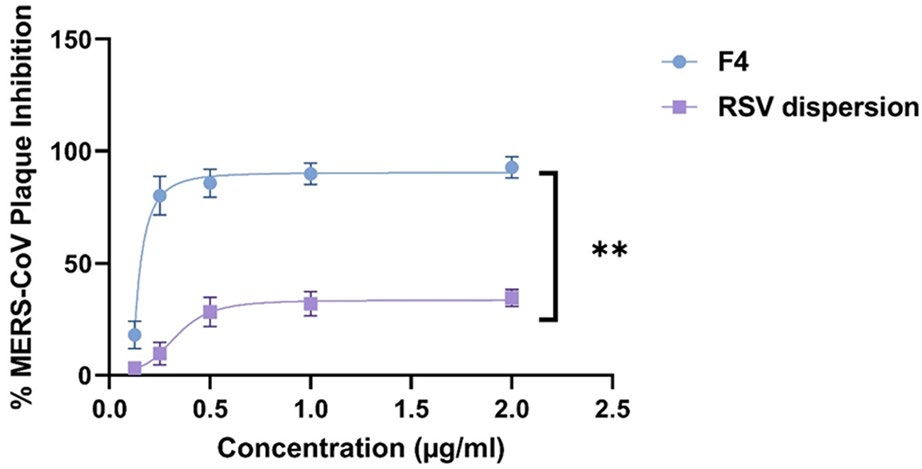
Plaque assay inhibition results of RSV dispersion (purple); RSV-loaded PEML (F4) (blue).

As a simple, reproducible and quantitative method, Plaque inhibition assays were implemented for determining the level of reduction in the (cytopathic activity) viral infectious capability via measuring the inhibition in cell culture formed plaques owing to the infection via serial dilutions of the virus. Figure 10 revealed viral plaque assays outcomes. The greatest viral plaque inhibition achieved at concentration of 2 µg/mL for both F4 was 92.7% ± 4.2%, while that of RSV dispersion was 34.6% ± 2.1%. The antiviral activity against Vero E6 cells challenged with MERS-CoV of F4 relative to that of RSV dispersion were (EC_50_ = 0.0127 µg/mL) and (EC_50_ = 0.34 µg/mL) respectively. These results denoted that RSV impart its antiviral activity even at diminished concentrations, moreover, this activity was significantly promoted (*p* < 0.01) on drug fabrication as Nano-vesicles. This boosted antiviral activity may be attributed to attachment and adherence of the drug loaded PEGylated nano-carriers to the viral cells permitting the drug accumulation surrounding the viral cells and creating a state of thermodynamically concentration gradient, hence RSV loaded lipid nano-carriers can diffuse easily through the viral membranes. To a great extent, it was previously reported that the composition of the lipidic nanovesicles as lipids and surfactants aid in the promotion of drug permeation to viral cellular membranes and serving the drug in solubilized form (Badria et al., 2020). In a more precise term Selectivity Index (SI) which governs both the efficacy of the drug in suppression of the viral multiplication relative to its cellular cytotoxicity and it was estimated utilizing SI = CC50/EC50 (Zakaria et al., 2021). SI value of F4 and RSV suspension were 724.4 and 7.3 affirming the significant superiority (*p* < 0.01) regarding selectivity of F4 relative to the RSV dispersion.

### 3.9. Pharmacodynamics assessments post infection

#### 3.9.1. Viral titer investigation in acutely infected lungs adopting plaque assay

**Figure 11.**
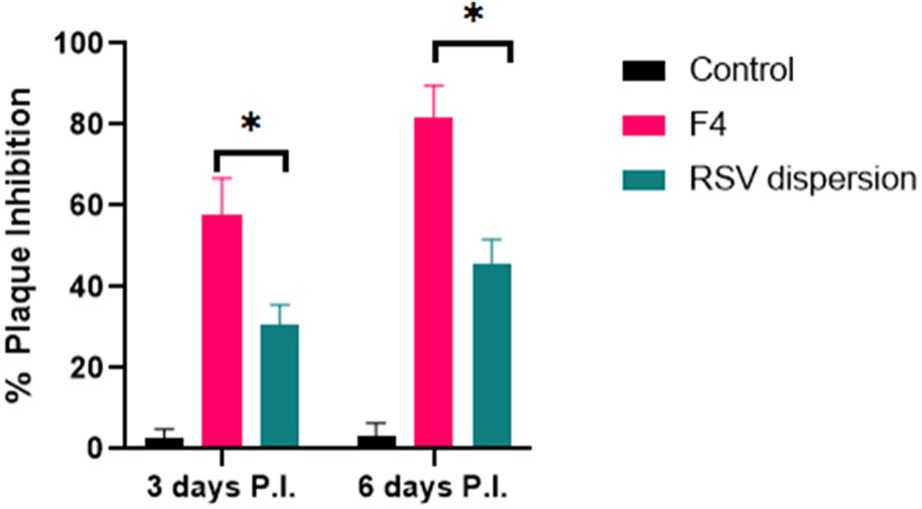
Impact of treatment with RSV-loaded formulation F4 relative to RSV dispersion at day 3 and day 6 postinfection on MERS-CoV titer via plaque assay (pfu/g lung homogenate ± SD).

The experiment timeline was started 6 h post infection and terminated day 6 post infection as previously mentioned that the virus required at least 6 h to be well recognized and noticed in the respiratory tract of the infected mice and on day 7 the virus could be barely detected (Iwata-Yoshikawa et al., 2019). Figure 11 revealed the % plaque inhibition values on day 3 and day 6 post infection in mice either treated by F4 57.6% and 82%, respectively, with prominent elevation relative to 30.5% and 45.6% of animals treated with RSV dispersion respectively. This may be attributed to the lipidic nature and diminished PS of F4 in contrast to RSV dispersion, which in turn promote the vesicular adhesion and penetration to virus cell, thus huge amount of RSV could be accumulated at and inside the virus cells, exerting prominent antiviral activity.

#### 3.9.2. The estimation of TNF-α, IL-6, SOD levels in BALF and serum level of GSH

**Figure 12.**
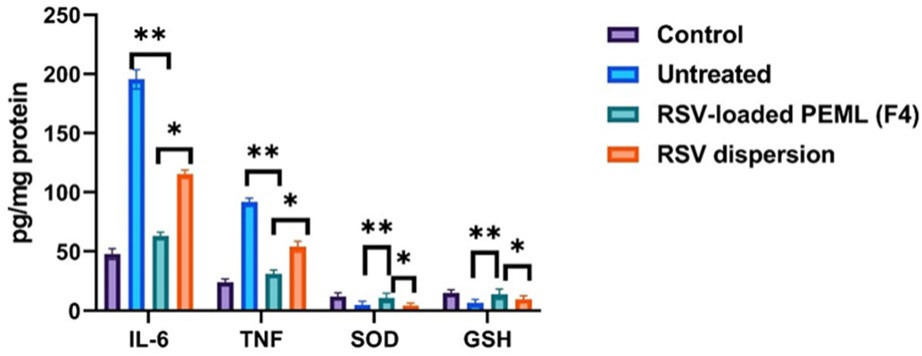
BALF levels of IL-6, TNF-α, SOD and plasma level of GSH ± SD in the experimental groups: control, untreated, infected group receiving the pure RSV dispersion and infected group receiving the RSV-loaded F4. * Denotes significance at *p* < 0.05; ** denotes significance at *p* < 0.01.

The significant(*p* < 0.05) elevation of TNF-α and IL-6 levels to 91.7 ± 9.8 and 195.5 ± 31.2 pg/mg protein, respectively after infection instead of 24.1 ± 5.30 and 48 ± 8.7 pg/mg protein respectively in the control group were observed denoting the huge inflammatory storm emergence associated with the infection. Meanwhile, their levels were suppressed to 54.1 ± 5.7 and 115.3 ± 37.2 pg/mg protein respectively after administration of RSV dispersion. Stunningly, the levels of TNF-α and IL-6 were significantly suppressed (*p* < 0.05) 31.3 ± 6.2 and 63.25 ± 8.1 respectively after administration of RSV loaded PEMLs (F4). In addition, 12.2 ± 1.3 was the level of SOD in BALF of control group and was significantly diminished to 5.05 ± 0.9 U/mg protein in viral infected groups. On another hand, the elevation of SOD 4.5 ± 1.2 and 11.1 ± 2.6 U/mg protein in the case of groups treated with RSV dispersion and F4, respectively could be depicted. Also the depletion in the serum levels of GSH to 6.5 ± 1.4 µM post viral infection instead of being 9.85 ± 2.3 µM in control group, while the treatment with RSV was able to restore GSH levels reaching to 9.6 ± 3.1 µM and 11.1 ± 2.2 µM in case of RSV dispersion and F4 respectively. These outcomes were in line with the findings that previously reported (Cong et al., 2018). Figure 12 illustrated the levels of IL-6, TNF-α, SOD and GSH levels in different groups: control, untreated, RSV dispersion and F4 receiving groups.

### 3.10. Pharmacokinetic study

**Figure 13.**
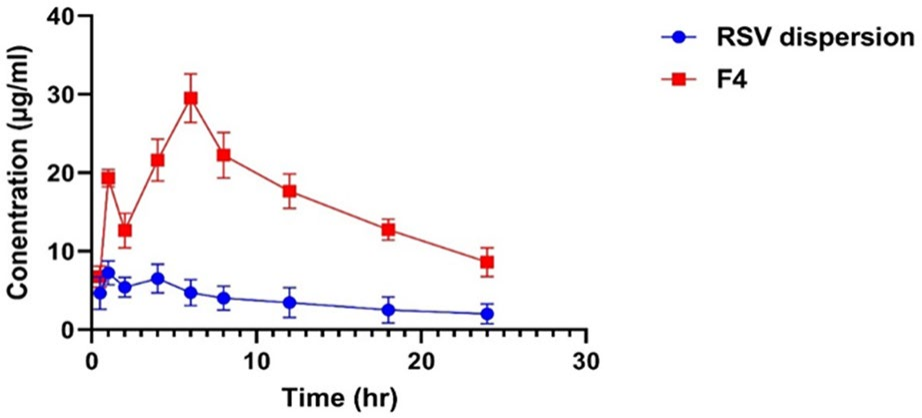
RSV plasma concentration at various time interval after oral administration of RSV-loaded PEML (F4) compared to that of RSV dispersion.

Figure 13 revealed the plasma concentration-time profiles of optimized RSV-loaded PEML (F4) relative to RSV dispersion. Table 3, sum up the pharmacokinetic parameters, it can be depicted that F4 parameters were significantly (*p* < 0.05) boosted than those of the RSV dispersion: The Cmax of F4 was 29.3 ± 2.8 µg/mL significantly (*p* < 0.05) higher than that of the RSV dispersion (7.2 ± 1.1 µg/mL). The Tmax value of 4 h for the F4 was two-fold higher than that of the RSV dispersion, likely owing to the extended liberation of RSV throughout the vesicular membrane. Moreover, F4 computed AUC _[0-24]_ was 397.2 ± 38.7 µg*h/mL which was superior significantly (*p* < 0.05) relative to RSV dispersion AUC _[0-24]_ 85.42 ± 21.3 µg *h/mL). The enhanced relative bioavailability of F4 relative to RSV suspension resulted from the capability of the lipidic carrier to invade the intestinal membranes, moreover the PEG sheath aid in extending the residence time in the circulation and granting the sustainability of its biological activity. From Figure 13 it can be depicted that the incidence of dual phases manifested by the evolution of two peak plasma drug concentrations: one at first hour and the other post four hour, owing to the well identified obstacles facing RSV as enterohepatic circulation and first pass metabolism following oral administration. Thus RSV concentration will be a consequence of enterohepatic circulation then reabsorption to blood and so on (Santos et al., 2019).

**Table 3.** Pharmacokinetic parameters for RSV post oral administration of the RSV-loaded PEML F4 compared with those of administered RSV dispersion.

Pharmacokinetic parameter	PEMLs formulation F4	RSV dispersion
C_max_ (µg/mL)	29.35 ± 2.8**	7.2 ± 1.1
T_max_ (h)	4.0**	2.0
AUC_0–24_ (µg h/mL)	397.2 ± 38.7**	85.42 ± 21.3
AUC_0–∞_ (µg h/mL)	537.3 ± 41.4	117.1 ± 18.1
Lz(hr^-1^)	0.044	0.058
T1/2 (h)	15.5	11.7
MRT	22	18.9

Abbreviations: AUC = area under the curve; PEMLs = PEGylated emulsomes.

Note: Data are expressed as mean ± SD for *n* = 6.

** Significant difference at (*p* < 0.05).

According to the outcomes acquired the ex-vivo and pharmacokinetics, it can be concluded that PEMLs was capable of resolve the pitfalls (constrained permeability and diminished aqueous solubility) that reside the bioavailability of RSV. Moreover, the formulation was able to boost the antiviral activity of RSV and in prohibiting the inflammatory storm and oxidative stress as consequences following the infection. Thus PEMLs can be claimed as potential panel to be included in the remedy protocol either in protection or resolving of the virus and the succeeded consequences (Figures 11–13).

## 4. Conclusion

Based on the study design which results in construction of eight formulations, the analysis of their related in-vitro characterizations: EE%, PS and ZP and picking F4 as an optimum formula according to the predetermined criteria of being of highest EE, ZP and minute PS, PEMLs can be successfully utilized as prosperous panels for oral delivery of RSV with boosted antiviral activity. Furthermore, the outcomes acquired the ex-vivo and pharmacokinetics, it can be concluded that PEMLs were capable of resolving the pitfalls (constrained permeability and diminished aqueous solubility) that reside the bioavailability of RSV. Moreover, the formulation was able to boost the antiviral activity of RSV and in prohibiting the inflammatory storm and oxidative stress as consequences following the infection. Thus PEMLs can be claimed as potential panel to be included in the remedy protocol either in protection or resolving of the virus and the succeeded consequences.

## Supplementary Material

Supplemental Material
