# Supplementary material for: Boosting the anti MERS-CoV activity and oral bioavailability of resveratrol via PEG-stabilized emulsomal nano-carrier: Factorial design, in-vitro and in-vivo assessments
Source: Drug Deliv. 2022 Sep 27;29(1):3155–67. doi: 10.1080/10717544.2022.2126028 (PMC9543103; doi:10.1080/10717544.2022.2126028)
Supplement: Supplemental Material [file IDRD_A_2126028_SM4941.docx]

**Supplementary Material**

**Material S 1:** Sigma-Aldrich, Inc., St. Louis, MO, USA, provided Resveratrol (RSV), cholesterol and Tristearin. (DSPE–mPEG-2000) was a generous gift from Lipoid GmbH (Ludwigshafen, Germany). BASF Co. provided Brij52 (polyoxyethylene (2) cetyl ether) (New Jersey, NY, USA). Gattefosse gave me Compritol 888 (glyceryl behenate) as a gift (France). El-Gomhouria Chemical Co., Cairo, Egypt, provided sodium chloride, potassium dihydrogen orthophosphate, methanol, sodium hydroxide, magnesium chloride, chloroform, and absolute ethanol. Spectrum Laboratories Inc., USA, provided the dialysis membrane (Spectra/Pore®, cut off 12,000-14,000). All chemicals and solvents were analytical grade and utilised exactly as they were given to us. ELISA kits that are specific (eBioscience Co, San Diego, CA, USA). Vero cells (African green monkey kidney cells, Vero C1008 [Vero 76, clone E6, Vero E6] were purchased from Viromed Laboratories (Minnetonka, MN, USA).

**Material S2:**

*Estimation of cytokines and oxidative biomarkers induced by MERS-CoV infection*

The animals were anesthetized by (1.6 g/kg) urethane and the blood and bronchial alveolar lavage fluid (BALF) were collected and prepared for further estimation of oxidative biomarkers and cytokines (Saadat et al., 2019). To prepare blood serum, 5ml blood was collected from the animal heart after opening the chest in the test tube and centrifuged at 3500 rpm for 10 min(Saadat et al., 2019). The serum samples were collected and stored at − 70 °C for measurement of the levels of total thiol content (GSH). Meanwhile in order to prepare BALF samples, according to a previous conducted study (Saadat et al., 2019), a cannula was located into the trachea and the right lung was washed with one mL normal saline for five times (totally, 5 ml) through a tracheal cannula. BALF was centrifuged at 2500 rpm at 4 °C for 10 min. The supernatant was collected and stored at − 70 °C for assessment of cytokines levels.

*2.8.3.1. Assessment of serum oxidant and antioxidant biomarkers*

Estimation of glutathione (GSH) rely on the fact that both protein and non-protein thiol (SH-) groups (mainly GSH) react with Ellman’s reagent [5,5’-dithiobis (2-nitrobenzoic acid)] to form a stable yellow color of 5-mercapto-2-nitrobenzoic acid, which can be measured colorimetrically at 412 nm (Shakeri et al., 2017).

*2.8.3.2. Assessment of IL-4 and TNF-α and SOD in BALF*

In order to estimate interleukin-6 (IL-4), *TNF-α, Superoxide dismutase (SOD)* precise ELISA kits (ebioscience Co, San Diego, CA, USA) was adopted and the instructions provided by the manufacturer were utilized. Statistical analysis was carried out by ANOVA followed by Tukey’s multiple comparisons test.

**Table S6.** Storage impact on the physical criteria of the optimum RSV-loaded PEMLs (F4).

| Parameter | Fresh F4 | Effect of storage on  F4 | Effect of storage on F4 |
| --- | --- | --- | --- |
|  |  | 4^o^C | 25^o^C |
| EE% | 75.8±3.7 | 73.63±3.4 | 72.2±2.6 |
| PS | 172.1±19.5 | 181.7 ± 18.5 | 185.3±11.4 |
| ZP | -43.6±7.3 | – 44.9 ± 0.65 | -47.1±3.8 |
